# Radiological findings in Behçet disease

**DOI:** 10.11604/pamj.2015.20.51.5928

**Published:** 2015-01-20

**Authors:** Chraa Mohamed, Kissani Najib, Lamia Essaadouni

**Affiliations:** 1 Neurology Department, Mohamed VI university hospital, Marrakesh, Morocco; 2 Internal medicine department, Mohamed VI university hospital, Marrakesh, Morocco

**Keywords:** Meuro-behçet disease, MRI, central nervous system, brainstem

## Abstract

Between 5 and 30% of patients with Behçet's disease will present neurological signs during the course of their illness. In order to evaluate the radiological signs on neuro-behçet disease, we studied consecutive patients in whom the diagnosis of this disease was retained, and who referred from January 2004 to December 2011 to the neurology and internal medicine departments in Mohammed VI universitary hospital in Marrakesh. Using 1.5T magnetic resonance imaging (MRI) with axial and coronal T2- weighted, axial Fluid Attenuated Inversion Recovery (FLAIR), pre- and post-contrast axial, coronal and sagittal T1-weighted sequences. The final number of patients in whom the diagnosis criteria of behçet disease were fulfilled and in whom an MRI was performed was 68 cases. Among these patients, 52 had parenchymal form of neuro-behçet with abnormalities in the MRI, 12 had vascular form and 4 patients had normal MRI. The brainstem, cerebral white matter, basal ganglia, internal capsule, thalamus and spinal cord were involved in forty four, thirty one, thirty, twenty nine, seventeen and four patients, respectively. The cerebral peduncle was the brainstem structure mainly involved with thirty cases followed by the pons with, twenty cases. Midbrain involvement interested forty patients. Brainstem atrophy was seen in eighteen cases. Finally, control MRI were obtained in four cases only, and showed changes in lesions size and shape.

## Introduction

Behçet´s Disease (BD) is a rare idiopathic chronic relapsing multisystem vascular-inflammatory disease. It was initially described by the Turkish dermatologist Dr. Hulusi Behçet in 1937 who described the triad of oral aphthous ulceration, genital ulcers and relapsing uveitis/iritis [1]. Other symptoms include CNS involvement, arthritis or arthralgia, gastrointestinal features, epididymitis, arterial occlusion or aneurysms, thrombophlebitis and deep vein thrombosis. Neurological involvement, which occurs in up to 49% of all cases, is often quite severe with a high rate of mortality and morbidity [2]. Radiologically, neuro-behçet disease (NBD) usually involves the white matter, brainstem, basal ganglia and thalamus [3]. Typical cerebral MRI often reveals T1 iso/hypointense and T2 hyperintense lesions [2, 4]. This study describes the pattern of radiological finding in NBD.

## Methods

Authors retrospectively studied the brain magnetic resonance imaging (MRI) findings of 68 patients diagnosed with NBD at the departments of neurology and internal medicine in Mohammed VI universitary hospital in Marrakesh from January 2004 to December 2011. The diagnosis of BD was performed by a senior neurologist or internal medicine physician, our patients fulfilled the criteria of the International Study Group for BD [1], the 68 patients included in this work all presented with neurological syndrome. Information extracted from the folders included age, gender, clinical form, neurological and extra neurological features, brain MRI finding as well as the clinical outcome and the MRI evolution of lesion after treatment if available (only 4 patients performed control MRI giving the lack of financial resources). Of the 68 patients, 4 patient´s MRI was normal but the NBD diagnosis was clinically certain, furthermore, cerebrospinal fluid (CSF) study was abnormal in all these 4 cases. Clinically, patients who had MRI abnormalities were divided into parenchymal and non-parenchymal groups, with 52 and 12 respectively. The course of the disease was either relapsing-remitting in 27 cases, progressive in 30 cases or monophasic in 11 cases. The MRI sequences included axial and coronal T2- weighted, axial Fluid Attenuated Inversion Recovery (FLAIR), pre- and post-contrast axial, coronal and sagittal T1-weighted sequences. Number; location; size; MR signal abnormalities; contrast enhancement; and presence of cerebral or brainstem atrophy were evaluated in patients with parenchymal involvement.

## Results

**Figure 1:**
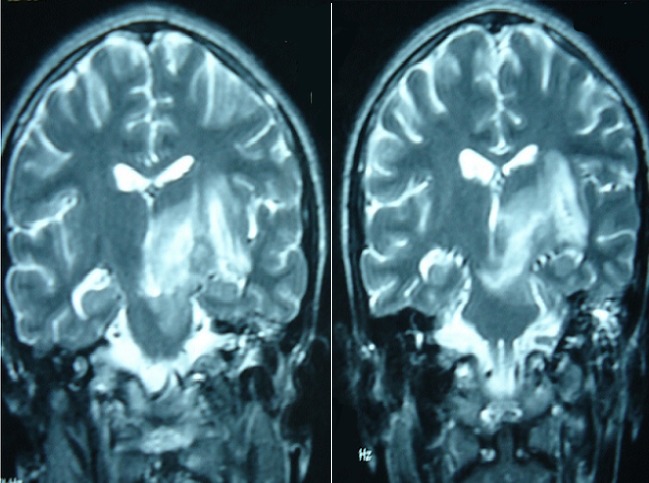
T2 weighted MRI sequences showing typical presentation of NBD with an association of brainstem and midbrain hypersignal lesions

**Figure 2:**
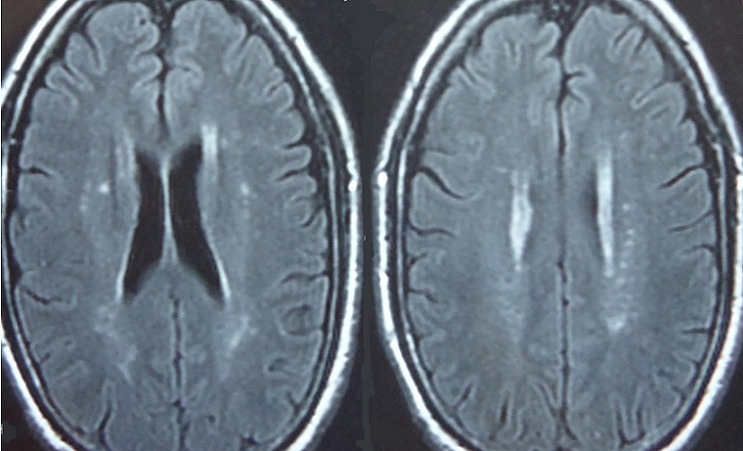
Flair weighted MRI sequences of two patients showing atypical presentations of neurobehçet disease

**Figure 3:**
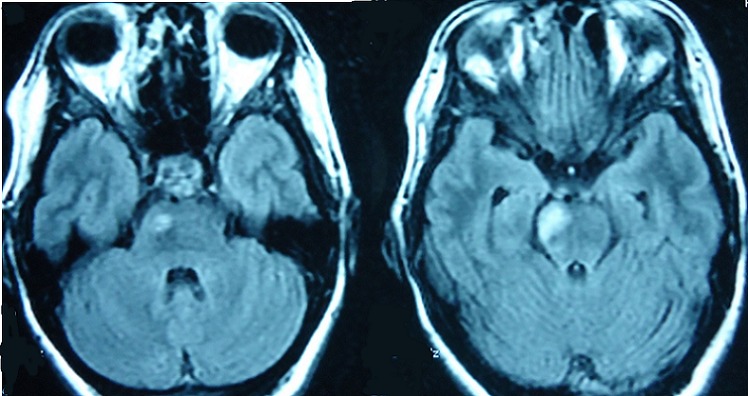
Flair weighted MRI sequences showing brainstem lesions with ventral localization

**Figure 4:**
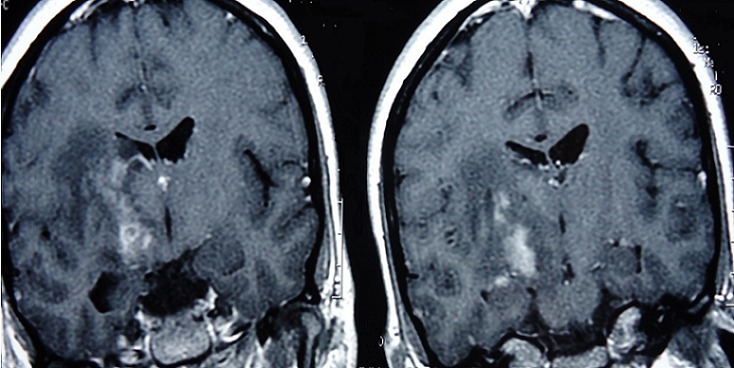
Pseudotumoral forms of neurobehçet disease

**Figure 5:**
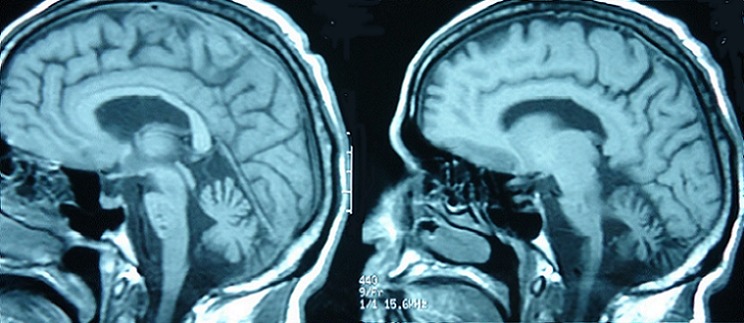
T1 weighted MRI sequences showing brainstem atrophy in a patient with NBD

In our series, 68 patients developed neurological symptoms related to BD. The main neurological symptoms revealing neurobehçet in our patients were in order of frequency; pyramidal signs, dysarthria, cerebellar signs with 49, 35, and 24 respectively, followed by other neurological symptoms (Table 1). Intracranial hypertension was the main neurological symptom in patients with extraparenchymal NBD. Other systemic manifestations included; oral in all patients, genital, ocular, dermatological, vascular and articular involvement (Table 2). The clinical presentation in our patients was monophasic in 11 cases, relapsing-remitting in 27 cases and progressive in 30 cases. Of all patients, we found 52 who presented with parenchymal MRI lesions, 12 who had extraparenchymal involvement (deep venous thrombosis), and in four cases the MRI was normal. Of those with parenchymal signs, the brainstem, cerebral white matter, basal ganglia, internal capsule, thalamus and spinal cord were involved in forty four, thirty one, thirty, twenty nine, seventeen and four patients, respectively (Table 3). Typically, the lesions were in hypersignal in T2 and FLAIR weighted MRI sequences, and in hyposignal T1 weighted sequences, they were not limited to one region but interested at least two structures, mainly an association of brainstem and midbrain structures (Figure 1). In 15 cases we found atypical MRI presentations like periventricular and white matter non specific lesions or confusing results when there is an association of vascular factors like hypertension (Figure 2). The brainstem lesions were ventrally located in thirty six up of the forty four patients who had MRI lesions in this region, and interested the cerebral peduncle in thirty cases followed by the pons with twenty cases (Figure 3). The lesions size was medium to large sized in the majority of cases, with pseudotumoral forms in three cases (Figure 4). In thirty three patients we had contrast enhancement after gadolinium injection, this was heterogeneous enhancement particularly present when the lesions are large. We also observed brainstem atrophy in eighteen cases and a cortico or/and subcortical one in six cases (Figure 5), we found these patients to have a chronic form in sixteen cases and a relapsing one in eight cases. In patients with extraparenchymal form, MR angiography which was performed in all patients confirmed the diagnosis. All our patients underwent a treatment associating intravenous bolus of corticoids (1 g per day for three days) in association cyclophosphamid (1 g per day for several months). The clinical evolution is summarized in Table 4. Four patients underwent MRI control after the treatment was stopped and showed improvement in lesions size and shape.

**Table 1:** Neurological symptoms in order of frequency

Neurological symptoms	
Pyramidal signs	49
dysarthria	35
Cerebellar signs	24
Bihavioral disorders	15
oculomotor deficit	14
ICH	13
sensitif signs	11
memory disorders	9
menigeal signs	8
epileptic seizures	6
Visuel deficit	3

**Table 2:** System organs involvement

Extraneurological involvement	
Buccal	68
Genital	52
Ocular	25
Dermatological	22
Vascular	15
articular	10

**Table 3:** Localisation of MRI lesions

Localisation of parenchymal lesions	
Brainstem	44
White matter	31
Basal ganglia	30
Internal capsule	29
Thalamus	17
Spinal cord	4

**Table 4:** Clinical evolution of our patients

Evolution	
Improvement	36
Stable	17
Worsning	8
Death	1
Lost to follow-up	6

## Discussion

BD is a systemic vasculitis which involves the central nervous system in up to 50% of cases [5], and is a devastating form in this case because of its high morbidities. Neurological symptoms are usually correlated with radiological findings. NBD can be separated into two forms; parenchymal and extraparenchymal (cerebro-vascular thrombosis) presentations. The extraparenchymal form occurs in up to 35% of cases [6], in our series we had 12 cases out of 68 (17.6%). MRI has a major role in asserting the diagnosis of neurobehçet disease as well as in understanding its physiopathology [7], especially because of the difficulties to obtain histopathological studies. In the parenchymal form of NBD, the lesions are usually multiple small foci of high intensity on T2 weighted sequences, although in our series we found them to be medium to large in the majority of cases. They are usually extensive, confluent and distributed over white matter without predilection for the periventricular regions in opposition to multiple sclerosis (MS) [8–11], besides in NBD the basal ganglia are involved which is atypical in MS. In another hand brainstem lesions we observed are ventrally localized, which was the case in other observation [12], in opposition to MS where the floor of the fourth ventricle and the middle cerebellar peduncle are frequently concerned [13], we observed an involvement of the later in only three cases. The lesions interests the white matter (70%), the brainstem (60%), the basal ganglia and the thalamus (40%) [13], in our series the brainstem was the first involved, followed by the white matter, but the majority of our patients had an association of brainstem and midbrain structure involvement. So we can say that MRI lesions of the mesocephalo-diancephalic junction are highly suggestive of NBD. Another differential diagnosis with NBD is lupus erythematous, here brainstem lesion can help differentiate between the two giving the fact that its involvement is rare in the last one [14], and the grey matter is more frequently involved in lupus and other vasculitis [13]. One remarkable finding is the frequency of contrast enhancement lesions in NBD (19 out of 52). Finally, the follow up showed a reversibility of lesions size in the four patients in whom a control MRI had been performed, which may reflect a reversible breakdown in the blood-brain barrier [15–18], this is inconsistent with other results where the authors had either an increase in lesion burden [7] or a lack of reversibility of the lesions [13].

## Conclusion

Although a retrospective study, our work offer results that are important in terms of MRI lesions characteristics in NBD (topography, size, contrast enhancement, atrophy). MRI was a powerful tool that help us in the diagnosis of NBD, the pattern of lesions found in this work can be highly suggestive of this diagnosis. Furthermore, the reversibility of the lesions in control MRI is a proof of the necessity to initiate an aggressive treatment when the diagnosis of NBD is suggested.
